# Accelerating Temperature-Dependent Optical Response Prediction in Truncated Pyramidal GaN/AlN Quantum Dots via Polynomial Ridge Regression

**DOI:** 10.3390/nano16211446

**Published:** 2026-11-08

**Authors:** Tesnim Brahim, Adel Bouazra, Beriham Ibrahim Basha, Fatma Aouaini

**Affiliations:** 1 Condensed Matter and Nanoscience Laboratory, Faculty of Sciences of Monastir, University of Monastir, Monastir 5019, Tunisia; adel.bouazra@isitc.u-sousse.tn; 2 Higher Institute of Computer Science and Communication Technologies of Sousse, University of Sousse, Sousse 4011, Tunisia; 3 Department of Physics, College of Science, Princess Nourah bint Abdulrahman University, P.O. Box 84428, Riyadh 11671, Saudi Arabia; bIbasha@pnu.edu.sa (B.I.B.); fasaidi@pnu.edu.sa (F.A.)

**Keywords:** polynomial ridge regression, quantum dots, temperature, leave-one-temperature-out cross-validation, optical response

## Abstract

A polynomial Ridge regression model is proposed to accelerate the prediction of temperature-dependent optical responses in a GaN/AlN truncated-pyramidal quantum dot. Reference transition energies were obtained at four temperatures (0, 100, 300, and 500 K) using a combination of coordinate-transformation and finite-difference methods. Leave-one-temperature-out cross-validation was used to select a second-order model with λ=0.178, yielding an NRMSE of 0.0335 and an $R^{2}$ of 0.9923. The predictions at 200 and 400 K remain independently unvalidated, whereas comparison with an independent FDM calculation at 600 K gives an absolute transition-energy error of 0.41 meV (0.35%). The reconstructed spectra exhibit the expected temperature-dependent shifts. An inference speed-up of up to $2.66\times 10^{7}$ was obtained for three additional temperatures, excluding model training and reference-data generation costs. These predictions have not been independently validated, and the small dataset limits conclusions regarding generalization. The approach is therefore presented as a preliminary surrogate demonstration for the investigated configuration.

## 1. Introduction

In recent years, nitride-based semiconductor quantum-dot (QD) heterostructures have attracted considerable research interest because of their distinct advantages over quantum wells [1,2] and quantum wires [3,4], including strong three-dimensional (3D) carrier confinement and enhanced optical and electronic properties. Group-III nitride materials, such as GaN, InN, and AlN, together with their ternary and quaternary alloys, can, in principle, cover a broad spectral range extending from the red to the ultraviolet region [5,6,7]. Continuous advances in GaN technology have therefore stimulated extensive research into the fabrication and characterization of both wurtzite (WZ) and zinc-blende (ZB) GaN/AlN quantum dots [8,9,10]. However, ZB GaN/AlN quantum dots remain less extensively studied than their WZ counterparts due to the metastable nature of the ZB phase in group-III nitrides, which poses significant challenges for the growth of high-quality epitaxial layers. Despite these challenges, zinc-blende GaN/AlN quantum dots remain of interest for optoelectronic applications because of their distinct structural and optical properties [11]. Compared with self-assembled WZ GaN quantum dots, the ZB phase can exhibit higher oscillator strengths and shorter radiative lifetimes, making it particularly attractive for high-performance optoelectronic and nonlinear optical devices [12,13]. In GaN/AlN heterostructures, the crystal structure plays a decisive role in determining the morphology of quantum dots. Zinc-blende GaN/AlN quantum dots typically adopt truncated pyramidal shapes with square bases. In contrast, wurtzite GaN/AlN quantum dots preferentially form truncated hexagonal pyramids, reflecting the hexagonal symmetry and polar nature of the wurtzite crystal structure [14,15]. Numerous studies have been devoted to the investigation of the nonlinear optical properties of GaN/AlN nanostructures. For example, Lu Zhang et al. [16] studied the effects of impurities and incident optical intensity on strained core–shell quantum dots (CSQDs). In another work, Keyin Li et al. examined the second-harmonic generation (SHG) coefficient of GaN/AlN quantum dots with tunable shapes under an applied electric field [17]. Moreover, A. Khaledi-Nasab et al. employed the finite element method to analyze the first and third-order absorption coefficients and refractive index variations in GaN/AlN quantum dots [18].

In the present work, a theoretical investigation is conducted to examine the influence of temperature on the electronic and optical properties of ZB GaN/AlN quantum dots. A coordinate-transformation technique combined with the finite-difference method (FDM) is employed to model truncated pyramidal quantum dots with square bases and to solve the corresponding three-dimensional Schrödinger equation. The coordinate-transformation approach was originally introduced by Creci and Inoshita [19,20] for modeling V-groove quantum wires and was subsequently extended to describe quantum-wire [21,22] and quantum-dot structures [23] with different geometries, heights, and lateral dimensions. More recently, Brahim et al. [24,25] demonstrated the effectiveness of combining coordinate-transformation and finite-difference methods with machine-learning algorithms to investigate the electronic and nonlinear optical properties of isolated and self-assembled semiconductor quantum dots. In these previous studies, machine-learning models were primarily employed to establish direct relationships between the considered structural or external parameters and selected electronic or optical quantities.

Recent studies have applied machine-learning models to the prediction of electronic and optical properties in semiconductor quantum nanostructures. For example, Ed-Dahmouny et al. employed supervised learning algorithms to directly predict specific optical coefficients under different external conditions [26,27].

In contrast, the present work presents a physics-guided strategy grounded in a polynomial Ridge surrogate. Unlike our previous studies, which directly mapped structural or external parameters onto selected electronic or optical properties, the present approach predicts the intersubband transition energy. This predicted energy is subsequently mapped into the density-matrix expressions to reconstruct the complete optical properties, including the total absorption coefficient and refractive-index change. The main novelty therefore lies in coupling a computationally inexpensive parametric surrogate with the underlying physical optical model. This hybrid framework reduces the need to repeatedly solve the three-dimensional Schrödinger equation and enables rapid interpolation of the optical response within the investigated temperature range.

The remainder of this paper is organized as follows. Section 2 presents the theoretical and numerical framework, including the temperature-dependent material parameters, the coordinate transformation, and the finite-difference discretization. Section 3 discusses the effects of temperature on optical properties and introduces the ridge regression model and evaluates its predictive performance. Finally, Section 4 summarizes the main conclusions and outlines possible extensions of the proposed approach.

## 2. Theoretical and Numerical Framework

To analyze the electronic and optical behavior of GaN/AlN quantum dots, a square truncated pyramid geometry is adopted, as shown in ref [28].

The quantized energy levels of electrons in the conduction band and their associated wave functions are determined using the three-dimensional Schrödinger equation [29].

(1) $$\begin{array}{cc} -\frac{\hbar^{2}}{2} & [\frac{\partial }{\partial x}\left(\frac{1}{m^{*}\left(x,y,z,T\right)}\frac{\partial \psi }{\partial x}\right)+\frac{\partial }{\partial y}\left(\frac{1}{m^{*}\left(x,y,z,T\right)}\frac{\partial \psi }{\partial y}\right)\\ & \;+\frac{\partial }{\partial z}\left(\frac{1}{m^{*}\left(x,y,z,T\right)}\frac{\partial \psi }{\partial z}\right)]+V\left(x,y,z,T\right)\psi =E\psi . \end{array}$$

Here, $m^{*}$ indicates the electron effective mass, V(x,y,z,T) is the three-dimensional potential profile presented by the conduction band offset, and *E* and ψ are the quantized energy levels and their corresponding wave functions, respectively. The conduction-band effective mass for the Γ valley, which depends on temperature, is taken from Refs. [14,30,31,32].

(2) $$\begin{array}{c} \frac{m_{0}}{m_{e}^{*}\left(T\right)}=1+2\gamma +\left[\frac{E_{p}^{\Gamma }\left(E_{g}^{\Gamma }+\frac{2}{3}\Delta_{s-o}\right)}{E_{g}^{\Gamma }\left(E_{g}^{\Gamma }+\Delta_{s-o}\right)}\right] \end{array}$$

Here, $m_{0}$ is the free electron mass, $\Delta_{s-o}=\left(0.017;0.019\right)\;\mathrm{eV}$ is the spin–orbit splitting energy, and γ=0 is the Kane parameter. $E_{p}^{\Gamma }=\left(25;27.1\right)\;\mathrm{eV}$ is the energy related to the momentum matrix element, for GaN and AlN, respectively.

$E_{g}^{\Gamma }$ is the energy gap, which is temperature dependent, and is given by Refs [33,34] as:

(3) $$E_{g}\left(T\right)=E_{g}\left(0\right)-\frac{\alpha T^{2}}{T+\beta },$$

$E_{g}\left(0\right)=\left(3.20;6.064\right)\;\mathrm{eV}$ is the energy gap at T=0 K, the Varshni temperature coefficients α=(0.77;2.65)meV/K and β=(600;2082)K for GaN and AlN, respectively.

Accordingly, under the effect of temperature, the confinement potential is expressed as:

(4) $$V\left(x,y,z,T\right)=\left\{\begin{array}{cc} 0, & \mathrm{inside}\;\mathrm{GaN}\;\mathrm{QD},\\ Q_{c}\;\left[E_{g}^{\Gamma }\left(\mathrm{AlN}\right)-E_{g}^{\Gamma }\left(\mathrm{GaN}\right)\right],\; & \mathrm{elsewhere}. \end{array}\right.$$

The conduction band discontinuity used in the modeling is assumed to constitute 70% of the band gap difference between GaN and AlN, consistent with reported heterostructure parameters [35].

The key step in the proposed numerical method is the construction of an appropriate coordinate-transformation function that accurately reproduces the geometry of the investigated quantum dot. Unlike the coordinate transformations employed in our previous studies [24,25,36], the present work introduces a new analytical transformation function specifically developed for the three-dimensional truncated pyramidal GaN/AlN quantum-dot geometry. This function enables the representation of the investigated geometry within a regular computational domain, facilitating the application of the finite-difference method. For a truncated pyramidal quantum dot with a square base, the corresponding geometrical function is defined as follows:

(5) $$\begin{array}{cc} x & =\beta .u,\\ y & =\beta \;f\left(u,v,w\right)=\frac{\beta }{k}\;\frac{\sinh(av)sech(dw)\;}{\cosh\;\left[u^{p}\tanh\left(nu^{q}\right)\right]},\\ z & =\beta .w. \end{array}$$

The coefficients β, *a*, *n*, *d*, *K*, *p*, and *q* are geometrical fitting parameters. They are adjusted to reproduce the actual dimensions, aspect ratio, sidewall curvature, and overall shape of the quantum dot. This transformation simplifies the geometry of the quantum dot, leading to flat interfaces in the computational domain. The first space corresponds to the real space defined by the old coordinates α(x,y,z), while the second space, γ(u,v,w), represents the computational space.

Consequently, the Schrödinger equation can be mapped into the γ-space as:

(6) $$\begin{array}{c} -\frac{\hbar^{2}}{2}\left[\begin{array}{cc} & u_{x}\frac{\partial }{\partial u}\psi^{1}\left(u,v,w\right)+v_{x}\frac{\partial }{\partial v}\psi^{1}\left(u,v,w\right)+w_{x}\frac{\partial }{\partial w}\psi^{1}\left(u,v,w\right)\\ & +u_{y}\frac{\partial }{\partial u}\psi^{2}\left(u,v,w\right)+v_{y}\frac{\partial }{\partial v}\psi^{2}\left(u,v,w\right)+w_{y}\frac{\partial }{\partial w}\psi^{2}\left(u,v,w\right)\\ & +u_{z}\frac{\partial }{\partial u}\psi^{3}\left(u,v,w\right)+v_{z}\frac{\partial }{\partial v}\psi^{3}\left(u,v,w\right)+w_{z}\frac{\partial }{\partial w}\psi^{3}\left(u,v,w\right) \end{array}\right]+V\psi =E\psi . \end{array}$$

Therefore, $\psi^{1}\left(u,v,w\right)$, $\psi^{2}\left(u,v,w\right)$, and $\psi^{3}\left(u,v,w\right)$ are functions, expressed as:

(7) $$\psi^{1}\left(u,v,w\right)=\left[\frac{u_{x}}{m^{*}}\frac{\partial \psi }{\partial u}+\frac{v_{x}}{m^{*}}\frac{\partial \psi }{\partial v}+\frac{w_{x}}{m^{*}}\frac{\partial \psi }{\partial w}\right].$$

(8) $$\psi^{2}\left(u,v,w\right)=\left[\frac{u_{y}}{m^{*}}\frac{\partial \psi }{\partial u}+\frac{v_{y}}{m^{*}}\frac{\partial \psi }{\partial v}+\frac{w_{y}}{m^{*}}\frac{\partial \psi }{\partial w}\right].$$

(9) $$\begin{array}{c} \psi^{3}\left(u,v,w\right)=\left[\frac{u_{z}}{m^{*}}\frac{\partial \psi }{\partial u}+\frac{v_{z}}{m^{*}}\frac{\partial \psi }{\partial v}+\frac{w_{z}}{m^{*}}\frac{\partial \psi }{\partial w}\right]. \end{array}$$

Then, we describe the two Jacobian matrices $J_{xyz}$ and $J_{uvw}$:

(10) $$J_{x,y,z}=J_{u,v,w}^{-1}$$

(11) $$J_{u,v,w}=\left[\begin{array}{ccc} x_{u} & x_{v} & x_{w}\\ y_{u} & y_{v} & y_{w}\\ z_{u} & z_{v} & z_{w} \end{array}\right]=\left[\begin{array}{ccc} \beta & 0 & 0\\ \beta f_{u} & \beta f_{v} & \beta f_{w}\\ 0 & 0 & \beta \end{array}\right]$$

(12) $$J_{x,y,z}=\left[\begin{array}{ccc} u_{x} & u_{y} & u_{z}\\ v_{x} & v_{y} & v_{z}\\ w_{x} & w_{y} & w_{z} \end{array}\right]=\left[\begin{array}{ccc} \frac{1}{\beta } & 0 & 0\\ -\frac{f_{u}}{\beta f_{v}} & \frac{1}{\beta f_{v}} & -\frac{f_{w}}{\beta f_{v}}\\ 0 & 0 & \frac{1}{\beta } \end{array}\right]=\frac{1}{\beta }\left[\begin{array}{ccc} 1 & 0 & 0\\ C & G & M\\ 0 & 0 & 1 \end{array}\right]$$

The derivatives of f(u,v,w) are $f_{u}$, $f_{v}$ and $f_{w}$:

For convenience, we define:

(13) $$C=-\frac{f_{u}}{f_{v}}$$

(14) $$G=\frac{1}{f_{v}}$$

(15) $$M=-\frac{f_{w}}{f_{v}}$$

Schrödinger equation becomes:

(16) $$\begin{array}{cc} -\frac{\hbar^{2}}{2\beta^{2}} & [\frac{\partial }{\partial u}\left(\frac{1}{m^{*}}\frac{\partial \psi }{\partial u}+\frac{C}{m^{*}}\frac{\partial \psi }{\partial v}\right)+\frac{\partial }{\partial v}\left(\frac{1}{m^{*}}\frac{\partial \psi }{\partial v}+\frac{C}{m^{*}}\frac{\partial \psi }{\partial u}\right)\\ & +\frac{\partial }{\partial v}\left(\frac{M}{m^{*}}\frac{\partial \psi }{\partial w}+\frac{G}{m^{*}}\frac{\partial \psi }{\partial v}\right)+\frac{\partial }{\partial w}\left(\frac{M}{m^{*}}\frac{\partial \psi }{\partial v}+\frac{1}{m^{*}}\frac{\partial \psi }{\partial w}\right)]+V\psi =E\psi . \end{array}$$

After discretizing the 3D equation using the finite difference method (FDM), we found:

(17) $$\begin{array}{cc} -\frac{\hbar^{2}}{2\beta^{2}} & [\left(L_{uu}^{1}{\left(\frac{1}{m^{*}}\right)}_{i+\frac{1}{2},j,k}-L_{uu}^{2}{\left(\frac{1}{m^{*}}\right)}_{i-\frac{1}{2},j,k}\right)\\ & +C_{i,j,k}\left(A_{uv}^{1}{\left(\frac{C}{m^{*}}\right)}_{i+\frac{1}{2},j,k}-L_{uv}^{2}{\left(\frac{C}{m^{*}}\right)}_{i-\frac{1}{2},j,k}\right)\\ \; & +C_{i,j,k}\left(L_{vu}^{1}{\left(\frac{1}{m^{*}}\right)}_{i,j+\frac{1}{2},k}-A_{vu}^{2}{\left(\frac{1}{m^{*}}\right)}_{i,j-\frac{1}{2},k}\right)\\ \; & +\left(L_{vv}^{1}{\left(\frac{C}{m^{*}}\right)}_{i,j+\frac{1}{2},k}-L_{vv}^{2}{\left(\frac{C}{m^{*}}\right)}_{i,j-\frac{1}{2},k}\right)\\ \; & +G_{i,j,k}\left(L_{vv}^{1}{\left(\frac{G}{m^{*}}\right)}_{i,j+\frac{1}{2},k}-L_{vv}^{2}{\left(\frac{G}{m^{*}}\right)}_{i,j-\frac{1}{2},k}\right)\\ \; & +M_{i,j,k}\left(L_{vw}^{1}{\left(\frac{M}{m^{*}}\right)}_{i,j+\frac{1}{2},k}-L_{vw}^{2}{\left(\frac{M}{m^{*}}\right)}_{i,j-\frac{1}{2},k}\right)\\ \; & +\left(L_{wv}^{1}{\left(\frac{M}{m^{*}}\right)}_{i,j,k+\frac{1}{2}}-L_{wv}^{2}{\left(\frac{M}{m^{*}}\right)}_{i,j,k-\frac{1}{2}}\right)\\ \; & +\left(L_{ww}^{1}{\left(\frac{1}{m^{*}}\right)}_{i,j,k+\frac{1}{2}}-L_{ww}^{2}{\left(\frac{1}{m^{*}}\right)}_{i,j,k-\frac{1}{2}}\right)]+V_{i,j,k}\psi_{i,j,k}=E_{i,j,k}\psi_{i,j,k}. \end{array}$$

The finite-difference coefficients are defined as follows:

(18) $$\begin{array}{cccc} L_{uu}^{1} & =\frac{\psi_{i+1,j,k}-\psi_{i,j,k}}{h_{u}^{2}},\; & L_{uv}^{1} & =\frac{\psi_{i+\frac{1}{2},j+\frac{1}{2},k}-\psi_{i+\frac{1}{2},j-\frac{1}{2},k}}{h_{u}h_{v}},\\ L_{vu}^{1} & =\frac{\psi_{i+\frac{1}{2},j+\frac{1}{2},k}-\psi_{i-\frac{1}{2},j+\frac{1}{2},k}}{h_{u}h_{v}},\; & L_{uu}^{2} & =\frac{\psi_{i,j,k}-\psi_{i-1,j,k}}{h_{u}^{2}},\\ L_{uv}^{2} & =\frac{\psi_{i-\frac{1}{2},j+\frac{1}{2},k}-\psi_{i-\frac{1}{2},j-\frac{1}{2},k}}{h_{u}h_{v}},\; & L_{vu}^{2} & =\frac{\psi_{i+\frac{1}{2},j-\frac{1}{2},k}-\psi_{i-\frac{1}{2},j-\frac{1}{2},k}}{h_{u}h_{v}},\\ L_{vv}^{1} & =\frac{\psi_{i,j+1,k}-\psi_{i,j,k}}{h_{v}^{2}},\; & L_{vw}^{1} & =\frac{\psi_{i,j+\frac{1}{2},k+\frac{1}{2}}-\psi_{i,j+\frac{1}{2},k-\frac{1}{2}}}{h_{v}h_{w}},\\ L_{wv}^{1} & =\frac{\psi_{i,j+\frac{1}{2},k+\frac{1}{2}}-\psi_{i,j-\frac{1}{2},k+\frac{1}{2}}}{h_{w}h_{v}},\; & L_{vv}^{2} & =\frac{\psi_{i,j,k}-\psi_{i,j-1,k}}{h_{v}^{2}},\\ L_{vw}^{2} & =\frac{\psi_{i,j-\frac{1}{2},k+\frac{1}{2}}-\psi_{i,j-\frac{1}{2},k-\frac{1}{2}}}{h_{v}h_{w}},\; & L_{wv}^{2} & =\frac{\psi_{i,j+\frac{1}{2},k-\frac{1}{2}}-\psi_{i,j-\frac{1}{2},k-\frac{1}{2}}}{h_{w}h_{v}},\\ L_{ww}^{1} & =\frac{\psi_{i,j,k+1}-\psi_{i,j,k}}{h_{w}^{2}},\; & L_{ww}^{2} & =\frac{\psi_{i,j,k}-\psi_{i,j,k-1}}{h_{w}^{2}}. \end{array}$$

In these equations, *i*, *j* and *k* denote the discretization-point indices, while $h_{u}$, $h_{v}$, and $h_{w}$ indicate the discretization steps along the *u*-, *v*-, and *w*-directions, respectively. $N_{u}$, $N_{v}$, and $N_{w}$ are the number of grid points in the corresponding directions. The discretization matrix is then constructed by imposing the following boundary conditions:

(19) $$\psi_{0,j,k}=\psi_{N_{u}+1,j,k}=0,\;\psi_{i,0,k}=\psi_{i,N_{v}+1,k}=0,\;\psi_{i,j,0}=\psi_{i,j,N_{w}+1}=0.$$

The transformed computational domain has dimensions of 10 × 2 × 10 nm and is discretized using a uniform grid spacing of 0.5 nm along u, v, and w. The same grid is used at all reference temperatures. Homogeneous Dirichlet boundary conditions are imposed on all six faces. The discretized eigenvalue problem is solved in double precision using the LAPACK routine DGEEV. This routine does not accept a user-defined convergence tolerance; its QR iteration uses internally controlled criteria tied to machine precision. Calculations were performed under Windows using a Fortran implementation developed in Developer Studio. The same coordinate-transformation and finite-difference approach has been applied to different quantum-confined geometries in our previous studies with quantitative comparisons against published experimental and theoretical optical transition data providing support for the framework [23,36].

**Figure 1:**
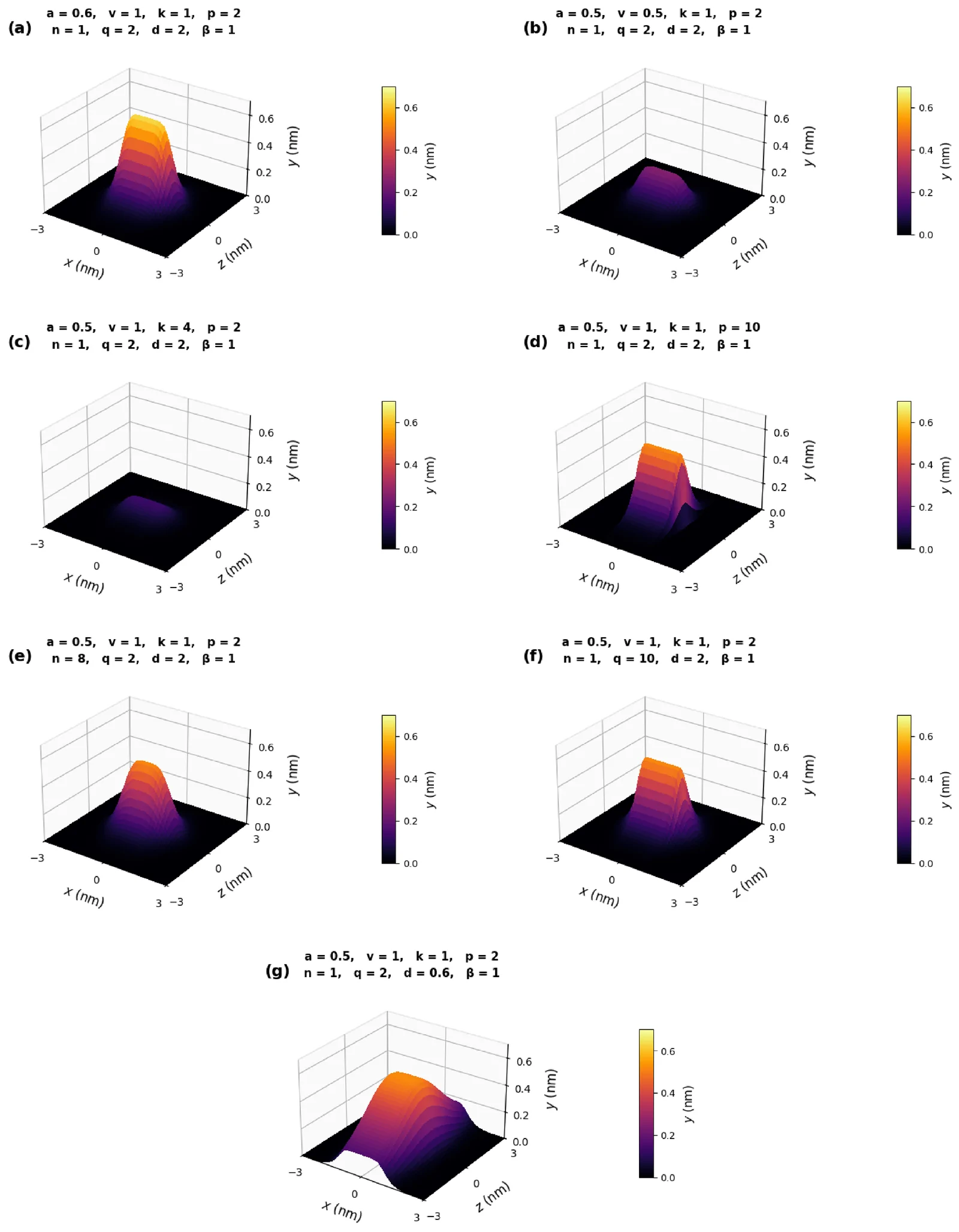
Impact of the transformation function parameters on the geometric dimensions of the quantum dot.

The morphological characteristics and spatial dimensions of the nanostructure are governed by the parameters *a*, *k*, *p*, *q*, *n*, *d*, and *v* as evidenced in Figure 1.

Figure 1 illustrates the influence of the fitting parameters of the coordinate-transformation function f(u,v,w) on the quantum dot morphology. For a fixed value of β=1 nm, the parameters *a*, *K*, and *v* primarily control the height, whereas *n*, *q*, *p*, and *d* govern the lateral dimensions and surface profile. By adjusting these parameters, different truncated-pyramidal geometries can be generated, with variations in height, lateral extension, sidewall steepness, and degree of truncation. This demonstrates the flexibility of the proposed approach in modeling quantum dots with different shapes and dimensions. The geometrical coefficients and grid dimensions are listed in Table 1.

## 3. Results and Discussion

### 3.1. Theoretical Method

This section presents a theoretical calculation of the optical properties of GaN/AlN quantum dots with truncated pyramidal cross-section.

In order to study the optical properties, the refractive-index change RIC and the absorption coefficient were calculated using the density-matrix formalism.

The first linear and third-order nonlinear (RIC) are given by Refs. [18,37,38]:

(20) $$\frac{\Delta n^{\left(1\right)}\left(\omega \right)}{n_{r}}=\frac{\sigma_{v}{\left|M_{21}\right|}^{2}}{2n_{r}^{2}\varepsilon_{0}}\left[\frac{E_{21}-\hbar \omega }{{\left(E_{21}-\hbar \omega \right)}^{2}+{\left(\hbar \Gamma_{12}\right)}^{2}}\right]$$

(21) $$\begin{array}{cc} \frac{\Delta n^{\left(3\right)}\left(\omega \right)}{n_{r}} & =-\frac{\sigma_{v}{\left|M_{21}\right|}^{2}}{4n_{r}^{3}\varepsilon_{0}}\frac{\mu cI}{{\left[{\left(E_{21}-\hbar \omega \right)}^{2}+{\left(\hbar \Gamma_{12}\right)}^{2}\right]}^{2}}\\ \; & \;\times [4\left(E_{21}-\hbar \omega \right){\left|M_{21}\right|}^{2}-\frac{{\left(M_{22}-M_{11}\right)}^{2}}{E_{21}^{2}+{\left(\hbar \Gamma_{12}\right)}^{2}}\\ \; & \;\times \left\{\left(E_{21}-\hbar \omega \right)\left[E_{21}\left(E_{21}-\hbar \omega \right)-{\left(\hbar \Gamma_{12}\right)}^{2}\right]-{\left(\hbar \Gamma_{12}\right)}^{2}\left(2E_{21}-\hbar \omega \right)\right\}] \end{array}$$

Then, the total (RIC) is written as:

(22) $$\frac{\Delta n(\omega ,I)}{n_{r}}=\frac{\Delta n^{\left(1\right)}\left(\omega \right)}{n_{r}}+\frac{\Delta n^{\left(3\right)}\left(\omega ,I\right)}{n_{r}}$$

The linear absorption coefficient (AC) is the following:

(23) $$\alpha^{\left(1\right)}=\left[\frac{\sigma_{v}\hbar \Gamma_{12}{\left|M_{21}\right|}^{2}}{{\left(E_{21}-\hbar \omega \right)}^{2}+{\left(\hbar \Gamma_{12}\right)}^{2}}\right]$$

The third-order nonlinear absorption coefficient (AC) is given by:

(24) $$\begin{array}{cc} \alpha^{\left(3\right)}\left(\omega ,I\right) & =-\omega \sqrt{\frac{\mu }{\varepsilon_{r}}}\left(\frac{I}{2\varepsilon_{0}n_{r}c}\right)\frac{\sigma_{v}\hbar \Gamma_{12}{\left|M_{21}\right|}^{2}}{{\left[{\left(E_{21}-\hbar \omega \right)}^{2}+{\left(\hbar \Gamma_{12}\right)}^{2}\right]}^{2}}\\ \; & \;\times \left\{4|M_{21}{|}^{2}-\frac{{\left(M_{22}-M_{11}\right)}^{2}\left[3E_{21}^{2}-4E_{21}\hbar \omega +\hbar^{2}\left(\omega^{2}-\Gamma_{12}^{2}\right)\right]}{E_{21}^{2}+{\left(\hbar \Gamma_{12}\right)}^{2}}\right\} \end{array}$$

The total (AC) can be expressed as:

(25) $$\alpha \left(\omega ,I\right)=\alpha^{\left(1\right)}\left(\omega \right)+\alpha^{\left(3\right)}\left(\omega ,I\right)$$

where $\sigma_{v}$ denotes the carrier density, $\Gamma_{12}$ is the relaxation rate between excited and ground states. $M_{ij}=\left|\langle \psi_{i}\;|\;e\vec{r}\;|\;\psi_{j}\rangle \right|$ is the electric dipole moment matrix element, $E_{21}=E_{2}-E_{1}$ is the transition energy between the two states and ℏω is the incident photon energy.

The corresponding parameters used in this work to model GaN/AlN QD are taken from these references, where $\Gamma_{12}=\frac{1}{T_{12}}$ is the relaxation rate between excited and ground states, with $T_{12}=0.1\;\mathrm{ps}$ [39,40], $\sigma_{v}=3\times 10^{22}\;m^{-3}$ [16,18,41], μ is the permeability of the system, $\varepsilon_{0}$ denotes the vacuum permittivity, and $\varepsilon_{r}$ denotes the real part of the relative permittivity, with $\varepsilon_{r}=n_{r}^{2}$ and $n_{r}=2.29$. Using the density matrix formalism, we focused on tailoring the linear, third-order, and total optical absorption coefficients and refractive index changes.

This study aims to further enhance the absorption coefficient under the effect of temperature. The calculations were performed for $I=0.1\;{\mathrm{MW}/\mathrm{cm}}^{2}$ and height H=4nm. Additional FDM calculations varying the conduction-band offset ratio around the adopted value of $Q_{c}=0.70$ showed that the blue shift between 0 and 500 K persists over the tested range. The investigation of temperatures up to 600 K is motivated by the high thermal stability of GaN/AlN materials. Previous experimental investigations by Nam et al. [42] examined the optical properties of GaN and AlN up to 700 and 800 K, respectively, while Patil and Melnik [43] theoretically investigated GaN/AlN quantum dots at temperatures reaching 1000 K. These studies support the physical relevance of exploring elevated temperatures. In the present model, temperature affects the bandgap, effective mass, and confinement potential, while the quantum dot geometry and material composition are assumed to remain unchanged. Therefore, the high-temperature results should be interpreted within these modeling assumptions.

**Figure 2:**
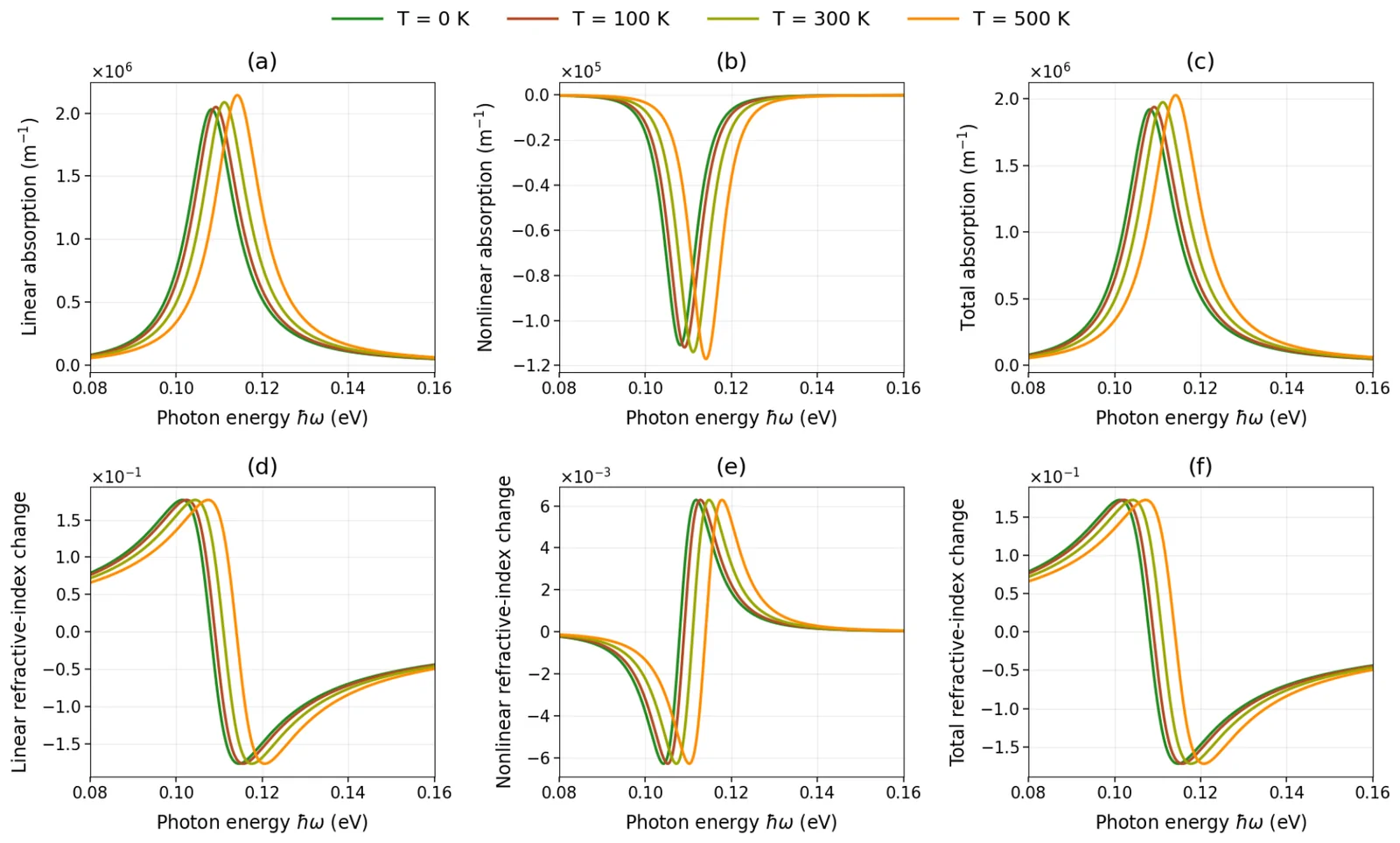
Variation of the optical response with temperature as a function of photon energy. (**a**) Linear (AC), (**b**) nonlinear (AC), (**c**) total (AC), (**d**) linear (RIC), (**e**) nonlinear (RIC), and (**f**) total (RIC).

As shown from Figure 2a–c for a given structural configuration, the linear absorption coefficient is positive, whereas the nonlinear contribution is negative, resulting in a reduction of the total absorption coefficient. An increase in temperature from 0 to 500K causes the total absorption coefficient to shift toward higher photon energies from 0.108 eV to 0.114 eV, while its amplitude slightly increases. This behavior is due to the reduction of the confinement potential, which in turn decreases the carrier effective mass. Since the absorption coefficients depend on the transition energy $E_{21}=E_{2}-E_{1}$ and the dipole matrix element $M_{ij}=\left|\langle \psi_{i}|er|\psi_{j}\rangle \right|$, both influenced by temperature, a competition between these parameters occurs, resulting in only a small increase in the absorption intensity. Figure 2d–f shows the refractive index changes as a function of temperature. A clear blue shift is observed with increasing temperature. The nonlinear temperature dependence of $E_{2}-E_{1}$ results from the coupled variations of the effective masses and conduction-band offset. The Varshni band gaps [Equation (3)] enter both the effective-mass expression [Equation (2)] and the confinement potential [Equation (4)]; they do not contribute additively to the intersubband transition energy. These changes affect the two confined levels differently. The calculated dipole matrix elements vary only slightly over the investigated temperature range and were therefore treated as constant in the spectral reconstruction.

### 3.2. Polynomial Ridge Regression

#### 3.2.1. Dataset Generation

The temperature-dependent transition energies used to build the surrogate model were obtained from the coordinate-transformation and FDM calculations. Therefore we considered four reference temperatures, T=0, 100, 300, and 500 K. For each temperature, the first two electronic energy levels were calculated, and the corresponding transition energy was determined.

*T* was used as the input variable, while the intersubband transition energy represented the regression target. The resulting initial dataset was therefore expressed as:

(26) $$D={\left\{\left(T_{i},\Delta E_{i}\right)\right\}}_{i=1}^{4}.$$

The relationship between temperature and transition energy was approximated using polynomial Ridge regression. For a polynomial of order *p*, the model is written as [44,45]:

(27) $$\hat{\Delta E}\left(T\right)=\beta_{0}+\sum_{j=1}^{p}\beta_{j}T^{j},$$

where $\beta_{j}$ denotes the regression coefficients. These coefficients were obtained by minimizing the regularized least-squares objective [45,46]:

(28) $$\hat{\beta }=\underset{\beta }{\arg\min}\left[\sum_{i=1}^{N}{\left(\Delta E_{i}-\hat{\Delta E}\left(T_{i}\right)\right)}^{2}+\lambda \sum_{j=1}^{p}\beta_{j}^{2}\right].$$

The regularization term was introduced to control the degree of regularization $\beta_{j}$ coefficient and reduce the risk of overfitting.

#### 3.2.2. Model Selection and Leave-One-Temperature-Out (LOTO) Validation

LOTO cross-validation was used for a limited number of reference temperatures. In each iteration, one temperature was completely held out from the training dataset, and the model was trained only on the three temperatures. The transition energy at the excluded temperature was then predicted and compared with the numerical value. The resulting held-out predictions were subsequently combined to calculate the performance metrics NMAE, NRMSE, NMSE and $R^{2}$ [47].

(29) $$\begin{array}{cc} \mathrm{NMAE} & =\frac{\frac{1}{N}\sum_{i=1}^{N}\left|\Delta E_{i}-{\hat{\Delta E}}_{i}\right|}{\Delta E_{\max}-\Delta E_{\min}}, \end{array}$$

(30) $$\begin{array}{cc} \mathrm{NRMSE} & =\frac{\sqrt{\frac{1}{N}\sum_{i=1}^{N}{\left(\Delta E_{i}-{\hat{\Delta E}}_{i}\right)}^{2}}}{\Delta E_{\max}-\Delta E_{\min}}, \end{array}$$

(31) $$\begin{array}{cc} \mathrm{NMSE} & =\frac{\frac{1}{N}\sum_{i=1}^{N}{\left(\Delta E_{i}-{\hat{\Delta E}}_{i}\right)}^{2}}{{\left(\Delta E_{\max}-\Delta E_{\min}\right)}^{2}}, \end{array}$$

(32) $$\begin{array}{cc} R^{2} & =1-\frac{\sum_{i=1}^{N}{\left(\Delta E_{i}-{\hat{\Delta E}}_{i}\right)}^{2}}{\sum_{i=1}^{N}{\left(\Delta E_{i}-\bar{\Delta E}\right)}^{2}}. \end{array}$$

For each polynomial degree, 121 logarithmically spaced values of λ between $10^{-12}$ and $10^{3}$ were evaluated. The degree–λ combination minimizing the aggregate four-fold LOTO NRMSE was selected, yielding degree 2 and λ≈0.178.

**Figure 3:**
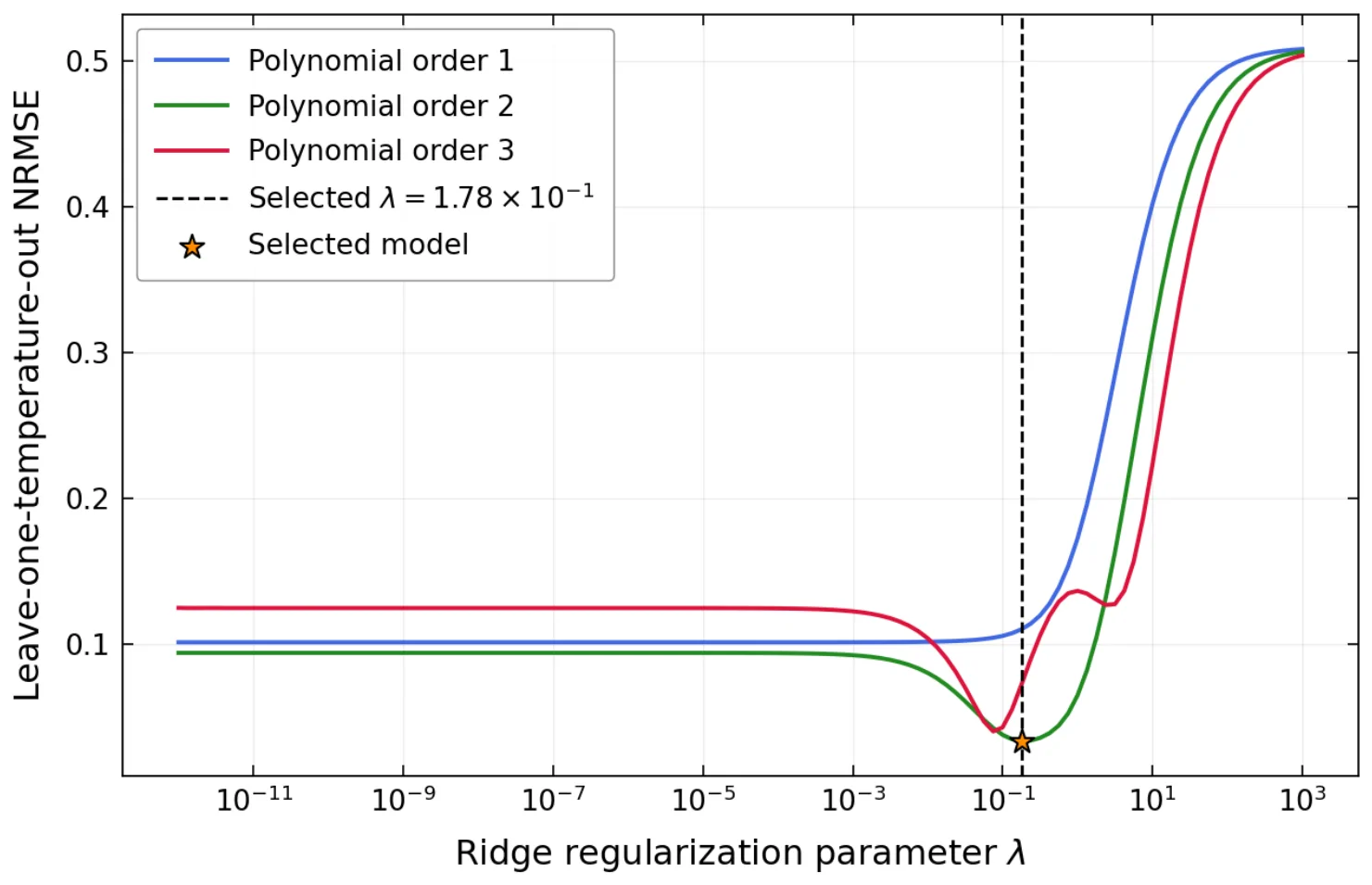
Selection of polynomial order and Ridge regulation parameter.

Figure 3 and Table 2 show that the second-order polynomial Ridge model with $\lambda =1.78\times 10^{-1}$ achieved the lowest validation NRMSE (0.033504), compared with 0.101180 and 0.040094 for the first- and third-order models, respectively. It also produced the lowest NMAE and NMSE and the highest coefficient of determination ($R^{2}=0.992303$). Therefore, the second-order model was retained for the subsequent temperature-dependent predictions.

With only three training temperatures per LOTO fold, the additional cubic term is weakly constrained and may increase prediction sensitivity. The quadratic model provides a simpler representation of the smooth temperature-dependent trend, yielding lower LOTO errors for the available dataset.

#### 3.2.3. Comparison with Second-Order Regression Models

To justify the choice of the proposed model, the selected second-order polynomial Ridge regression was compared with unregularized polynomial regression and second-order polynomial Lasso regression. All models used the same quadratic representation defined previously (Equation (26)) and the same LOTO cross-validation protocol. The unregularized model estimates its coefficients by minimizing the residual sum of squares, whereas Lasso introduces an $L_{1}$ penalty, $\lambda \sum_{j=1}^{2}\left|\beta_{j}\right|$. In contrast, Ridge employs an $L_{2}$ penalty, $\lambda \sum_{j=1}^{2}\beta_{j}^{2}$ [46,48].

As shown in Table 3, the second-order polynomial Ridge model achieves the lowest LOTO NMAE, NRMSE, and NMSE, and the highest $R_{\mathrm{LOTO}}^{2}$ (0.992303) among the quadratic models considered. These results support its selection for subsequent temperature-dependent predictions within the investigated configuration. Individual LOTO predictions and absolute errors are reported in Table 4, while the fitted coefficients and regularization parameters are provided in Table 5. These results should be interpreted in the context of the four available reference temperatures. Each LOTO fold uses only three training points, and the folds at 0 and 500 K involve extrapolation beyond the corresponding training range. Moreover, selecting the polynomial order and λ using the same LOTO results employed for performance assessment may introduce optimism into the reported metrics. The high $R^{2}$ supports the ability to capture the smooth temperature-dependent trend for the investigated configuration, while broader generalization requires independent validation at additional temperatures.

#### 3.2.4. Comparison with Conventional Interpolation Methods

To compare the predictive performance of polynomial Ridge regression with conventional interpolation methods, linear interpolation, natural cubic spline, and piecewise cubic Hermite interpolating polynomial (PCHIP) were evaluated using the same leave-one-temperature-out (LOTO) procedure. At each iteration, one temperature was excluded, and the remaining three observations were used for fitting. For the boundary temperatures (0 and 500 K), predictions involved extrapolation beyond the training interval using the corresponding methods. As shown in Table 6, the second-order Ridge model achieved the lowest NRMSE (0.033504) and the highest $R^{2}$ (0.992303). However, these results should be interpreted cautiously given the limited number of reference temperatures and the precision of the reported energies.

#### 3.2.5. Prediction of Intersubband Transition Energy

**Figure 4:**
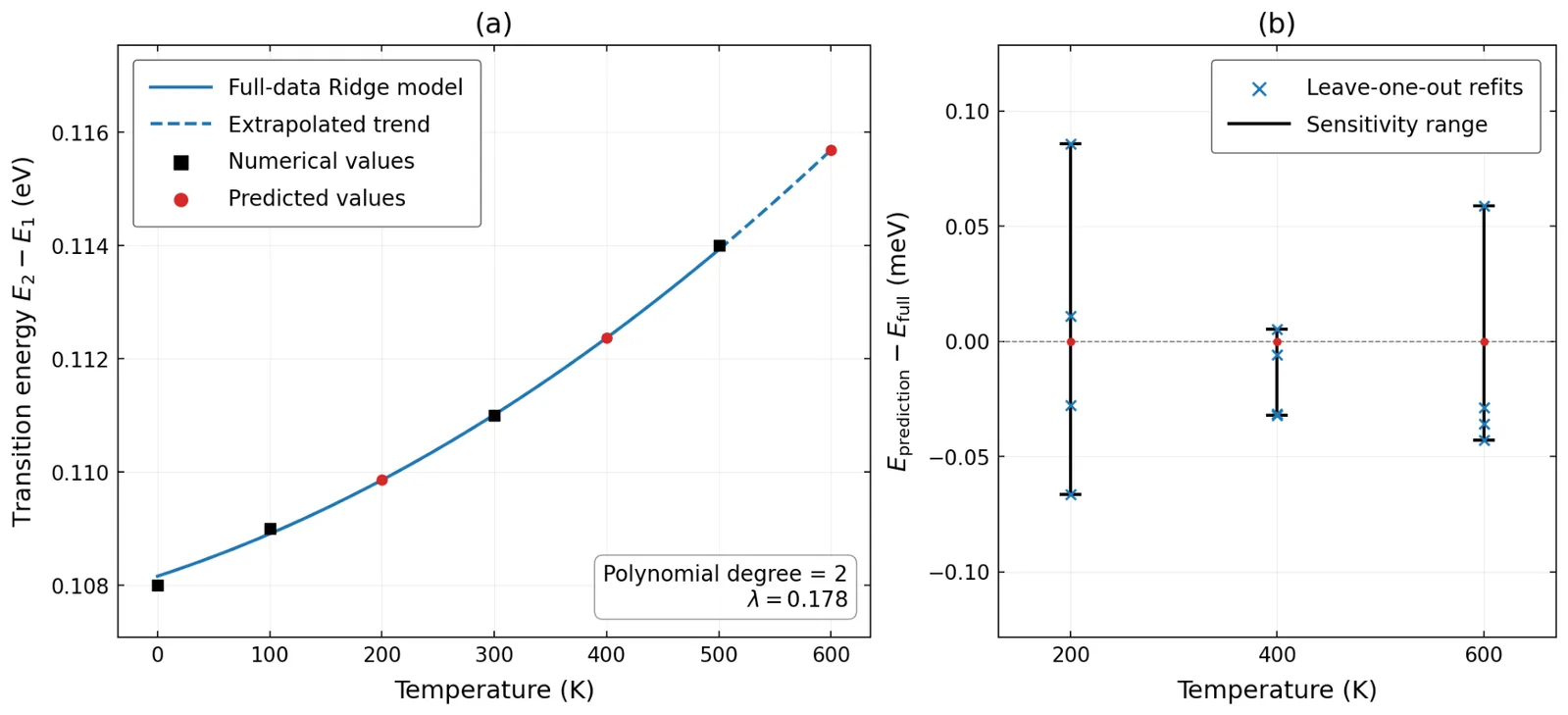
Reference and Ridge-predicted transition energies. (**a**) Temperature dependence, including FDM-validated extrapolation at 600 K. The dashed blue line represents the extrapolation from 500 to 600 K. (**b**) Sensitivity to omitting individual training temperatures, with degree 2 and λ=0.178. Bars show prediction ranges, not confidence intervals.

After model selection, the second-order polynomial Ridge model was retrained using all four reference temperatures to predict the transition energies at 200, 400, and 600 K. Figure 4a shows the progressive increase in the predicted transition energy with temperature. The estimates at 200 and 400 K correspond to interpolation, whereas the estimate at 600 K corresponds to extrapolation. The predictions at 200 and 400 K remain to be independently validated. Figure 4b assesses sensitivity to training-data selection. The model was refitted after omitting each reference temperature in turn, with degree 2 and λ=0.178 held fixed. Bars span the minimum and maximum deviations from the full-data prediction across all five fits. These ranges describe training-data sensitivity, not confidence or prediction intervals, and exclude numerical discretization and physical-model uncertainty.

An independent numerical method calculation at 600 K yields a transition energy of 0.1161 eV, compared with the Ridge prediction of 0.1156903 eV, corresponding to an absolute error of 0.41 meV (0.35%). This point was excluded from model training and selection, providing an independent assessment of extrapolation at this temperature. The observed discrepancy exceeds the sensitivity range, confirming that this range measures training-data sensitivity rather than predictive uncertainty. These ranges also exclude the effects of rounding the reference energies.

#### 3.2.6. Prediction of the Optical Properties

**Figure 5:**
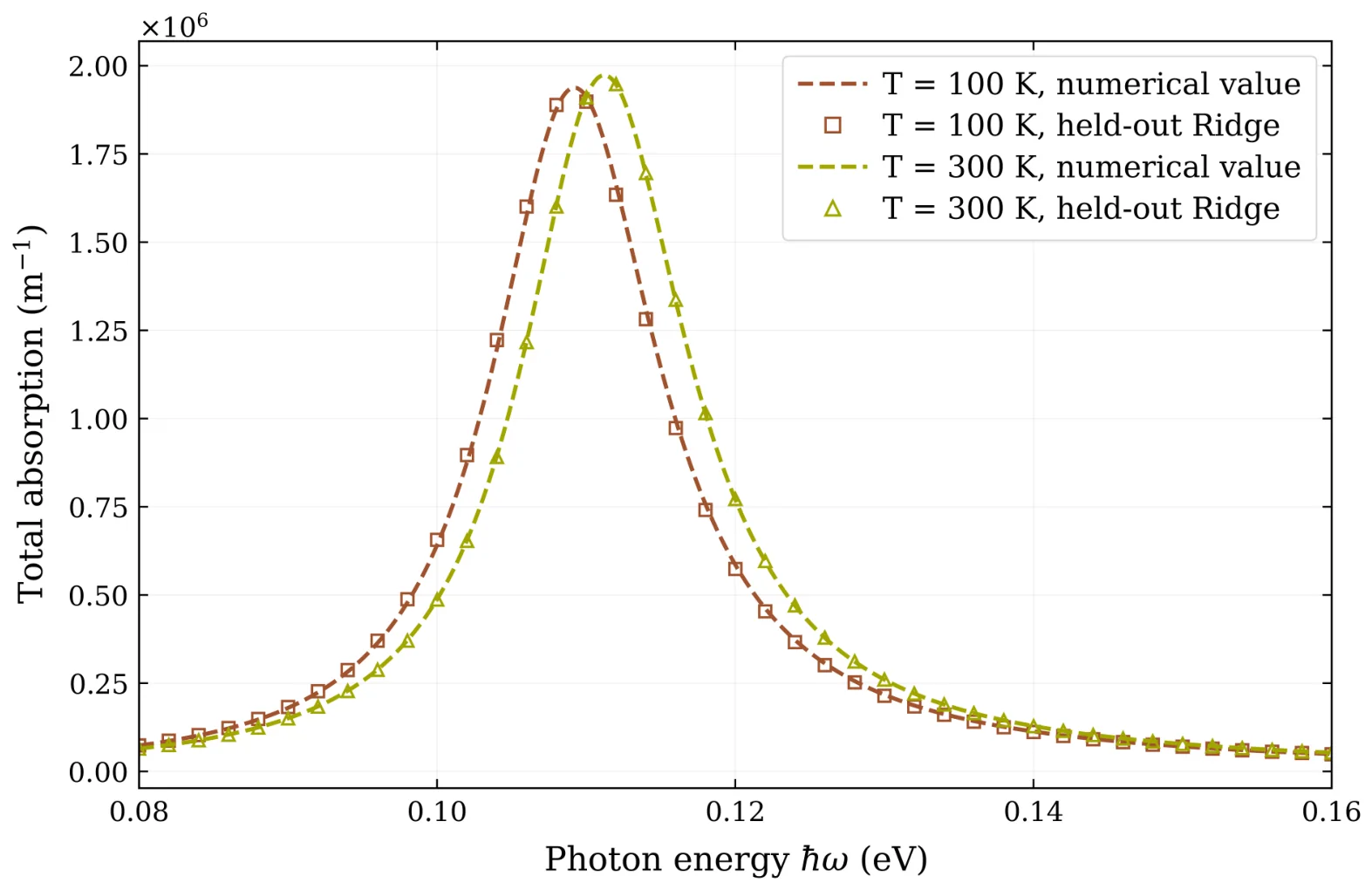
Comparison between the numerical and held-out Ridge predictions of the total AC at T = 100 K and T = 300 K.

**Figure 6:**
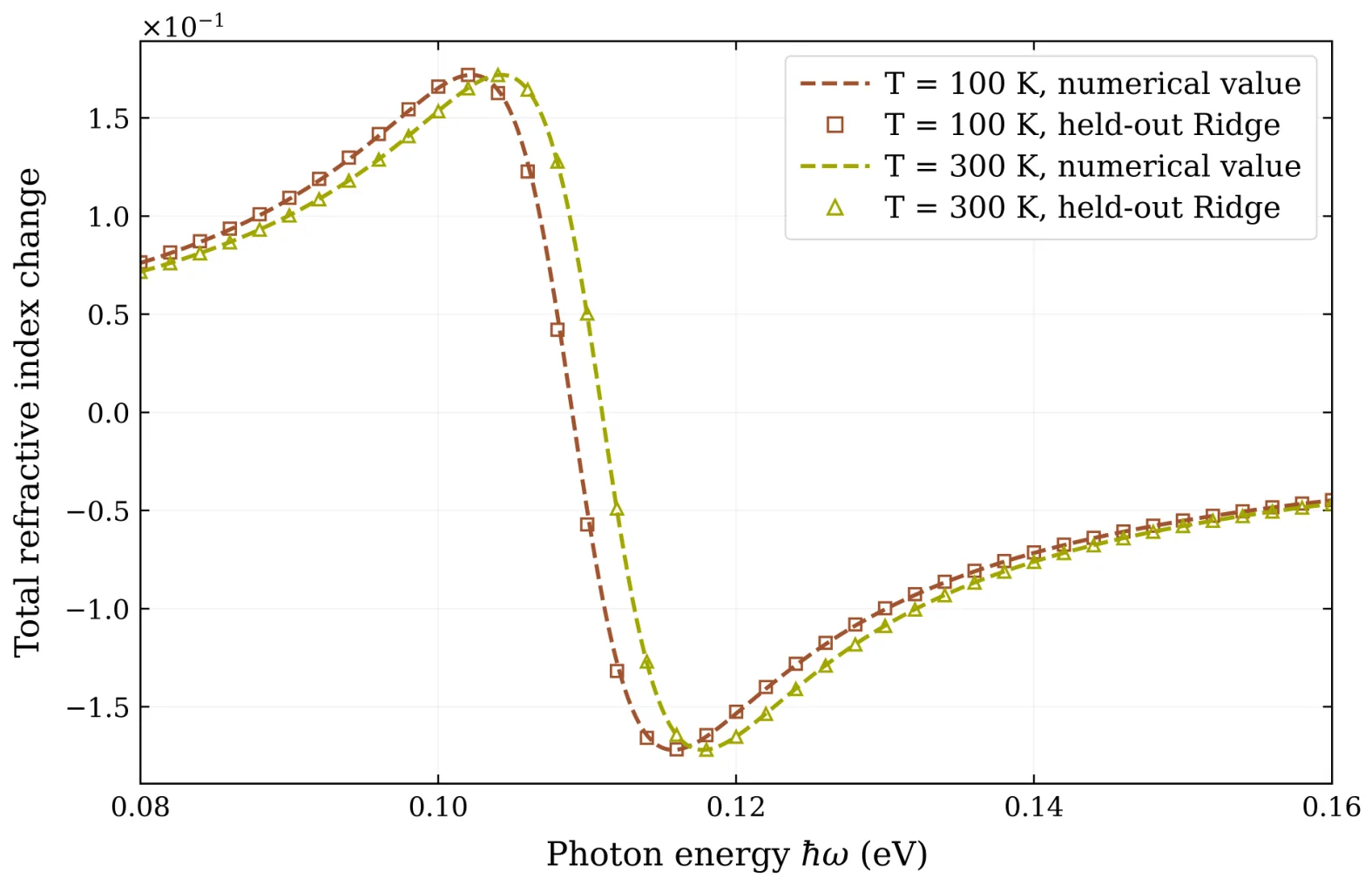
Comparison between the numerical and held-out Ridge predictions of the total RIC at T = 100 K and T = 300 K.

At new temperatures, the Ridge-predicted transition energy is inserted into the optical-response expressions, while the dipole-related inputs are held at their reference values. This constant-dipole approximation avoids additional Schrödinger-equation calculations for spectral reconstruction. Figure 5 and Figure 6 compare the total absorption coefficient and refractive-index change reconstructed from held-out Ridge predictions with the numerical reference curves at 100 and 300 K. The shift toward higher photon energies with increasing temperature is consistent with the increase in the intersubband transition energy. The close agreement between the corresponding curves indicates that the predicted transition energies preserve the main spectral features at these held-out temperatures.

To further assess the accuracy of the Ridge-based spectral reconstruction, a quantitative comparison was performed at the held-out temperatures of 100 and 300 K, considering peak position, peak magnitude, integrated absorption, and curve-wise RMSE. For the total absorption coefficient, the peak position errors were 0.1 and 0.0 meV, the relative peak magnitude errors were 0.1489% and 0.0125%, and the curve-wise RMSE values were 11625.02 and 1126.63 m^−1^, respectively. The corresponding integrated absorption errors were 0.1535% and 0.0138%. For the total refractive-index change, the peak position errors were 0.2 and 0.0 meV, the relative peak magnitude errors were 0.000845% and 0.000745%, and the curve-wise RMSE values were 0.002058 and 0.000196, respectively. These results indicate close agreement between the reference spectra reconstructed from numerical transition energies and the Ridge-based predictions.

**Figure 7:**
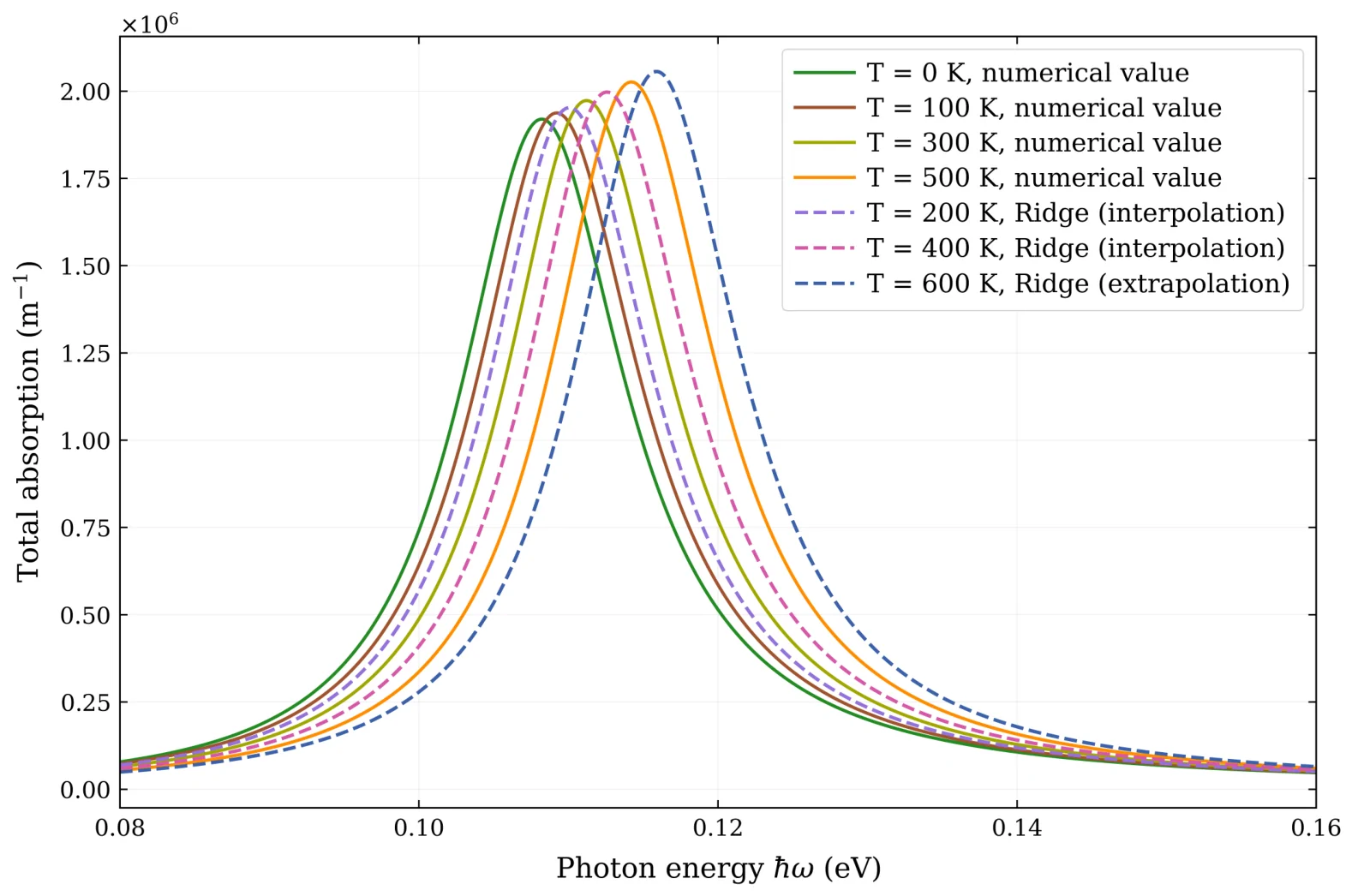
Predicted total optical absorption coefficient at 200, 400, and 600 K using the quadratic Ridge regression model. The results at 600 K represent extrapolation beyond the reference temperature range (0–500 K).

**Figure 8:**
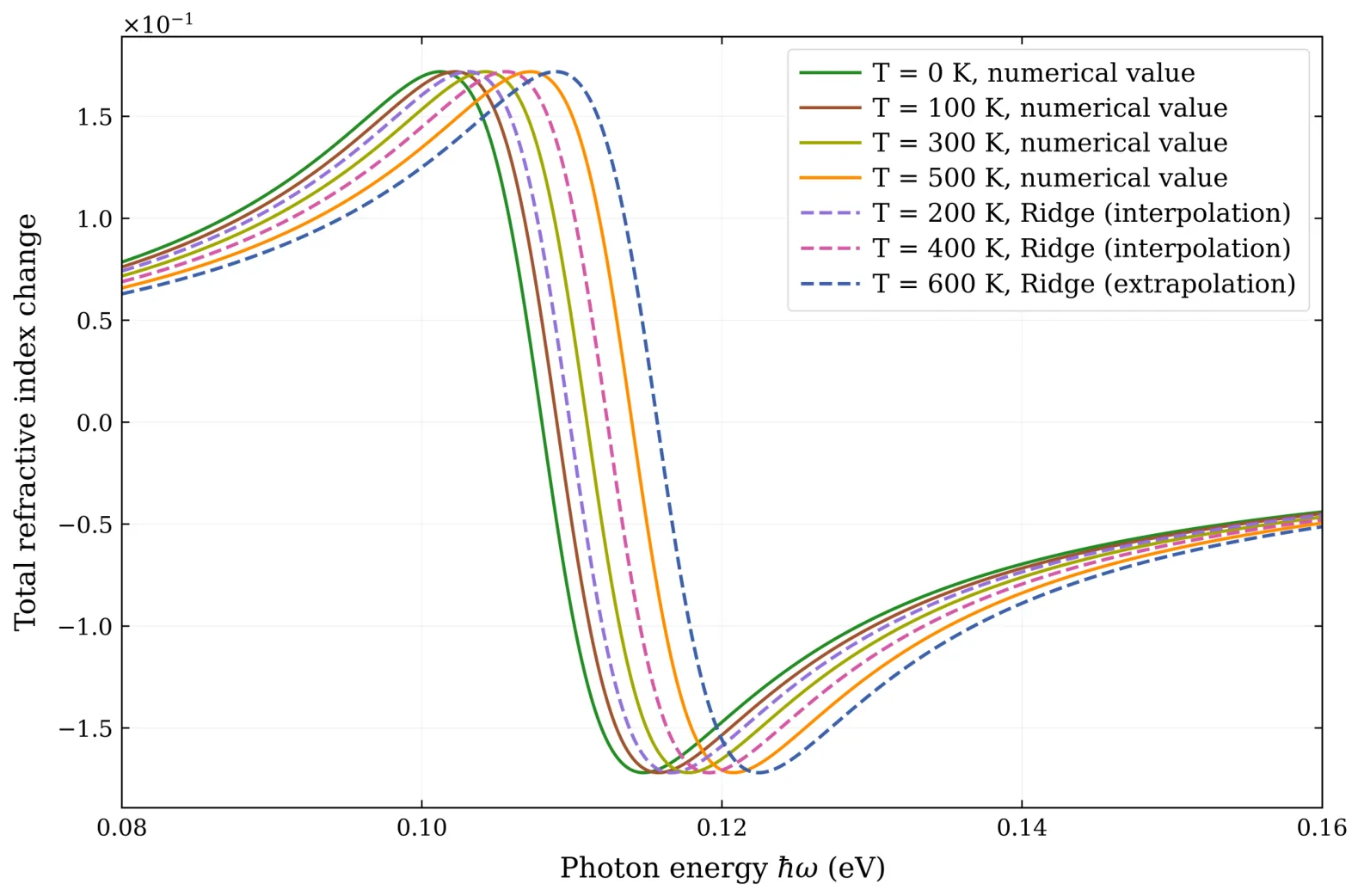
Predicted total refractive-index change at 200, 400, and 600 K using the quadratic Ridge regression model. The results at 600 K represent extrapolation beyond the reference temperature range (0–500 K).

Figure 7 and Figure 8 illustrate the variation of total AC and RIC respectively as a function of temperature using the optimized Ridge regression model. As the temperature increases, both response curves exhibit a progressive shift toward higher photon energies, consistent with the increase in the intersubband transition energy. The predicted curves at 200, 400, and 600 K show a smooth evolution relative to the numerical reference results.

#### 3.2.7. Computational Efficiency of the Ridge Regression Model

The numerical simulation time was estimated at approximately 45 min (2700 s) per temperature. Thus, the four reference calculations required to construct the training dataset correspond to approximately 180 min. Using these reference data, the training of Ridge model required only 0.001295 s. Once trained, the model evaluated the optical response (total RIC and AC) at one additional temperature in 0.000207 s, whereas a vectorized evaluation at three additional temperatures required 0.000305 s. These computation times are summarized in Table 7.

These values correspond to the median computation times obtained over 30 runs and exclude plotting and file input/output operations. Compared with the estimated coordinate transformation combined with FDM computation times, the Ridge model achieved inference speed-up factors of $1.30\times 10^{7}$ and $2.66\times 10^{7}$ for one and three additional temperatures, respectively.

The coordinate transformation and FDM calculations were performed under Windows on an AMD Ryzen 7 7730U processor with 16 GB RAM. Ridge timings were obtained in Google Colab under Linux using an Intel Xeon CPU at 2.20 GHz and 12.67 GiB RAM, with Python 3.13.16, NumPy 2.1.3, and scikit-learn 1.6.1. The speed-up ratios compare estimated FDM runtimes with measured surrogate evaluation times on different computing environments and exclude reference-data generation and model training. Including the 180 min required to generate the four reference points, the surrogate workflow becomes computationally advantageous over direct numerical calculations (45 min per temperature) from five additional temperature evaluations. For the three additional temperatures considered here, this threshold is not reached. The reported speed-ups concern inference only.

To highlight the contribution of the present study, Table 8 compares our approach with previous investigations [24,25] in terms of input variables, predicted outputs, validation strategies, and main contributions. While Ref. [24] focuses on the number of coupled quantum dots and Ref. [25] investigates alloy composition using leave-one-composition-out (LOCO) validation, the present work evaluates the prediction of intersubband transition energies at previously unseen temperatures using leave-one-temperature-out (LOTO) cross-validation. The predicted transition energies are then used to reconstruct the corresponding optical absorption and refractive index change spectra using a compact theoretical formalism.

## 4. Conclusions

The choice of coordinate-transformation function provides an effective representation of the truncated-pyramidal geometry by mapping the GaN/AlN quantum dot onto a regular computational domain. Combined with the finite-difference method, this transformation enables the solution of the three-dimensional Schrödinger equation and the analysis of temperature-dependent electronic and optical responses. Building on these numerical results, the optimized second-order polynomial Ridge model captures the temperature-dependent transition-energy trend, as assessed through LOTO cross-validation, and enables reconstruction of the corresponding absorption and refractive-index-change spectra at a low inference cost. However, since the model is based on only four reference temperatures and a single input variable, these results should be regarded as a preliminary surrogate demonstration for the investigated configuration. For the available dataset, the quadratic Ridge model yields the lowest LOTO errors among the models considered. The predictions at 200 and 400 K remain independently unvalidated, whereas the transition-energy prediction at 600 K was assessed against an independent FDM calculation, giving an absolute error of 0.41 meV (0.35%). The proposed framework combines geometrical adaptability and rapid prediction, offering a promising approach for the investigated GaN/AlN quantum-dot configuration. Future work will focus on expanding the reference dataset and validating the interpolated and extrapolated predictions through additional independent numerical calculations. The framework could also be extended to other quantum-dot shapes and dimensions, as well as additional external physical parameters.

## Figures and Tables

**Table 1:** Geometrical and numerical parameters used in the finite-difference calculations.

Parameter	Value or Setting
*a*	0.408
*k*	0.1
*n*	0.98
*d*	1.1
p,q	2,2
β	1 nm
Computational domain	10×2×10 nm
Grid spacing	$h_{u}=h_{v}=h_{w}=0.5$ nm
Grid points (u,v,w), including boundaries	21×5×21
Boundary conditions	Homogeneous Dirichlet on all six faces
Eigensolver	LAPACK DGEEV
Numerical precision	Double precision
Convergence tolerance	Internal LAPACK criteria; no user-specified tolerance
Implementation	Fortran, Developer Studio under Windows

**Table 2:** Optimized performance of the polynomial Ridge models using LOTO cross-validation.

Order	λ	NMAE	NRMSE	NMSE	$R_{\mathrm{LOTO}}^{2}$
1	$1.00\times 10^{-12}$	0.082942	0.101180	0.010237	0.929800
2	$1.78\times 10^{-1}$	0.026284	0.033504	0.001123	0.992303
3	$7.50\times 10^{-2}$	0.035588	0.040094	0.001608	0.988977

**Table 3:** LOTO performance comparison of second-order polynomial regression models.

Model	NMAE	NRMSE	NMSE	$R_{\mathrm{LOTO}}^{2}$
Polynomial regression	0.078125	0.093935	0.008824	0.939494
Polynomial Lasso	0.078125	0.093935	0.008824	0.939494
Polynomial Ridge	0.026284	0.033504	0.001123	0.992303

Regularization parameters were selected separately using LOTO: λ=0.177827941 for Ridge and $\lambda =10^{-12}$ for Lasso. The latter lies at the lower boundary of the tested range and yields nearly unregularized predictions. The same folds informed selection and performance assessment; the reported metrics may therefore be optimistic.

**Table 4:** Individual LOTO predictions (eV) and absolute errors (meV), given as prediction/absolute error.

Model	0 K	100 K	300 K	500 K
Reference ΔE (eV)	0.108	0.109	0.111	0.114
Polynomial Ridge, order 1	0.107583/0.417	0.109026/0.026	0.111548/0.548	0.113000/1.000
Polynomial Ridge, order 2	0.108355/0.355	0.108842/0.158	0.111015/0.015	0.113897/0.103
Polynomial Ridge, order 3	0.108404/0.404	0.108832/0.168	0.110858/0.142	0.113860/0.140
Polynomial regression, order 2	0.108375/0.375	0.108800/0.200	0.111300/0.300	0.113000/1.000
Polynomial Lasso, order 2	0.108375/0.375	0.108800/0.200	0.111300/0.300	0.113000/1.000

Each held-out temperature was excluded from its corresponding fit. Errors were calculated before rounding predictions for display. Reference energies are available to three decimal places in eV; additional prediction digits do not imply equivalent physical precision.

**Table 5:** Coefficients of models fitted to all four reference temperatures, with energy in eV and temperature in K.

Model	Order	λ	$\beta_{0}$	$\beta_{1}$	$\beta_{2}$	$\beta_{3}$
Polynomial Ridge	1	$10^{-12}$	0.10783050847	$1.1864406780\times 10^{-5}$	–	–
Polynomial Ridge	2	0.17782794100	0.10815650436	$6.4718171570\times 10^{-6}$	$1.0140991799\times 10^{-8}$	–
Polynomial Ridge	3	0.074989420933	0.10812673701	$7.1232487304\times 10^{-6}$	$5.8546578994\times 10^{-9}$	$6.7516197729\times 10^{-12}$
Polynomial regression	2	–	0.10806030151	$7.3743718593\times 10^{-6}$	$8.9195979899\times 10^{-9}$	–
Polynomial Lasso	2	$10^{-12}$	0.10806030151	$7.3743718567\times 10^{-6}$	$8.9195979847\times 10^{-9}$	–

The units of $\beta_{j}$ are $\mathrm{eV}\;K^{-j}$, whereas $\beta_{0}$ is expressed in eV. The coefficients were converted from standardized polynomial features to temperature units. Additional digits are reported for numericalreproducibility and should not be interpreted as physical precision. For Lasso, the minimum LOTO NRMSE was obtained at the lower boundary of the investigated regularization range, $\lambda =10^{-12}$, resulting in predictions nearly identical to those of unregularized polynomial regression.

**Table 6:** Comparison of second-order polynomial Ridge regression and conventional interpolation methods using leave-one-temperature-out (LOTO) cross-validation.

Method	NMAE	NRMSE	NMSE	$R^{2}$
Polynomial Ridge (order 2)	0.026284	0.033504	0.001123	0.992303
Natural cubic spline	0.065712	0.089329	0.007980	0.945283
Linear interpolation	0.062500	0.093169	0.008681	0.940476
PCHIP interpolation	0.079682	0.095178	0.009059	0.937882

**Table 7:** Comparison of the computation times between numerical method and ridge regression ML model.

Quantity	Numerical Method	Ridge	Inference Speed-Up
Model training (s)	–	0.001295	–
One additional temperature (s)	2700	0.000207	$1.30\times 10^{7}$
Three additional temperatures (s)	8100	0.000305	$2.66\times 10^{7}$

**Table 8:** Comparison of previous studies and the present work.

Criteria	Ref. [24]	Ref. [25]	Present Study
QD system	Coupled GaN/AlN QDs	Truncated-pyramidal GaN/AlGaN QDs	Truncated-pyramidal GaN/AlN QD
Input variables	Number of coupled QDs	Photon energy and alloy composition	Temperature
Outputs	Transition energy and nonlinear optical rectification	Optical absorption coefficient	Transition energy, optical absorption, and refractive index change
Methods	Polynomial regression, Ridge, Lasso, Elastic Net	ANN, Random Forest, Decision Tree, KNN, polynomial regression	Polynomial regression, Ridge, Lasso, linear interpolation, cubic spline, PCHIP
Validation	Comparison with FDM results	Random 80/20 split and LOCO	LOTO cross-validation
Contribution	Fast prediction for larger coupled QD systems	Composition-dependent optical absorption prediction	Unseen-temperature prediction and optical response reconstruction

## Data Availability

The numerical simulation datasets generated during this study are available from the corresponding author upon reasonable request.
